# Lack of association of amyloid beta progression with cognitive changes in marmosets with PSEN1 mutations

**DOI:** 10.1002/alz70855_104844

**Published:** 2025-12-24

**Authors:** Lauren Bailey, Leila Letica, Takeshi Murai, Lauren Mongeau, Andrew DeSana, Lauren R Dubberley, Ryan Robert Dyer, Diego Szczupak, David J Schaeffer, Lauren K Hayrynen Schaeffer, Tingting Zhang, Annat Haber, Catrina Spruce, Seung‐Kwon Ha, Julia Oluoch, Brianne Stein, Sang‐Ho Choi, Gregory W Carter, Afonso C Silva, Stacey J Sukoff Rizzo

**Affiliations:** ^1^ University of Pittsburgh School of Medicine, Pittsburgh, PA, USA; ^2^ The Jackson Laboratory for Genomic Medicine, Farmington, CT, USA; ^3^ The Jackson Laboratory, Bar Harbor, ME, USA; ^4^ University of Maine, Orono, ME, USA; ^5^ Tufts University Graduate School of Biomedical Sciences, Boston, MA, USA

## Abstract

**Background:**

Fundamental questions remain about the key mechanisms that initiate Alzheimer's disease (AD), the factors that promote its progression, and the relationship with cognitive impairment. Therapeutic interventions have been successful at clearing amyloid plaque accumulation, though fail to rescue and prevent cognitive decline. To understand the association between amyloid burden and cognition decline, we are studying non‐human primates that carry a C410Y point mutation in the presenilin‐1 (*PSEN1*) gene that confers early onset AD in human carriers, and the association of cognitive function with amyloid progression measured by fluid and neuroimaging biomarkers.

**Method:**

Marmosets carrying PSEN1 mutations and age‐and sex‐matched non‐carrier controls are studied longitudinally from birth throughout their lifespan. Beginning at 6‐weeks of age and at 6‐month intervals, plasma biomarkers of amyloid‐beta (Aβ1‐40, Aβ1‐42), total tau, neurofilament light chain (NfL), and glial fibrillary acidic protein (GFAP) were analyzed. Marmosets were trained on a cognitive battery that was delivered annually using a touchscreen including tests of spatial working memory and reversal learning (delayed‐match and delayed‐non‐match‐to‐position), recognition memory (delayed‐match‐to‐sample), a serial reaction time task to measure attention and a progressive ratio task to measure motivation. Amyloid uptake was measured with 11C‐PiB‐PET.

**Results:**

Beginning at 12‐months of age PSEN1 marmosets demonstrate significant increases in plasma Aβ42:40 relative to non‐carriers. PSEN1 marmosets were indistinguishable from non‐carriers in task acquisition across the cognitive testing battery with no significant impairment in measures of visual and spatial working memory capacities, attention, or motivation up to 2 years of age. When compared to age and sex matched unmanipulated WT control marmosets, PSEN1 marmosets have similar 11C‐PiB uptake values, indicative of little to no difference between cortical amyloid burden.

**Conclusion:**

Increases in brain and plasma amyloid beta do not correlate with cognitive changes in young PSEN1 carrier animals, indicating a lack of direct association between amyloid progression and cognition. Longitudinal study of these marmosets provides the opportunity to study the earliest primate‐specific mechanisms that contribute to the molecular and cellular root causes of cognitive decline associated with early onset AD and disease progression.

